# Ferrets and genetically modified ferrets as model organisms in biomedical research: a review

**DOI:** 10.3389/fgeed.2026.1777289

**Published:** 2026-07-01

**Authors:** Hongshu Sui, Yiyang Li, Yan Yang, Liangyu Jiang, Yaxin Jiao, Dongwei Liu, Haoyu Liu, Zekai Yang, Haorui Chen, Dongyu Zhang, Ruihan Ma, Yuxin Jiang, Mingjiu Luo

**Affiliations:** 1 Shandong Provincial Key Laboratory for Livestock Germplasm Innovation and Utilization, College of Animal Science and Technology, Shandong Agricultural University, Tai’an City, Shandong, China; 2 Department of Histology and Embryology, School of Clinical and Basic Medicine, Shandong First Medical University and Shandong Academy of Medical Science, Jinan, Shandong, China; 3 Morphology Laboratory, School of Clinical and Basic Medical Sciences, Shandong First Medical University and Shandong Academy of Medical Sciences, Jinan, China

**Keywords:** animal model, basic and translational research, ferret, genetically modified, respiratory infection

## Abstract

Domestic ferrets have served as experimental animals for over a century, offering a balance between practicality and biological relevance while bridging the gap between rodent and non-human primate models. Their anatomical and physiological similarities to humans, particularly in the respiratory and nervous systems, make ferrets an invaluable model for biomedical research across various disciplines. Recent advances in genome-editing technologies have enabled the efficient creation of genetically modified (GM) ferrets, significantly expanding their applications in basic and translational research. This review describes the unique biological characteristics of ferrets and their current applications in modeling human pathogenic infections, respiratory, gastrointestinal, and neurological disorders, as well as other complex diseases that rodent models fail to effectively recapitulate. We also discuss the challenges associated with ferret models and outline the future directions to enhance their utility, with the goal of advancing our understanding of human diseases and supporting the development of novel therapeutics.

## 1 Introduction

Ferrets (*Mustela* [*M.*] *putorius furo*), a member of the *Mustelidae* family, were domesticated from polecats (*M. putorius, M. eversmanii*) more than 2,000 years ago. Historically, ferrets were used to hunt rabbits and rodents, and they have long been valued as domestic pets (Thomson, 1951). The first documented use of domestic ferrets in biomedical research dates back to 1911 (Yeates, 1911). Today, ferrets are best known for their critical role in studying respiratory viruses, particularly the influenza virus. However, their utility in biomedical research has also been extended to a diversity of other fields, including anatomy, neurology, endocrinology, reproductive physiology, emerging viral diseases, vaccine development, and modeling human diseases that are not effectively recapitulated by rodent models (Pramod et al., 2024).

The growing demand for ferrets, both in research and as pets, has led to large-scale commercial breeding. Marshall Farms in North Rose, New York, US, which has bred ferrets for more than 80 years, is now the largest supplier of ferrets (Marshall Ferret®) for scientific research purposes in North America. Specific pathogen-free (SPF) ferrets are bred and raised within barrier facilities at Marshall BioResources under strict conditions to ensure the health and welfare of the animals (Navratil, 2016).

**FIGURE 1:**
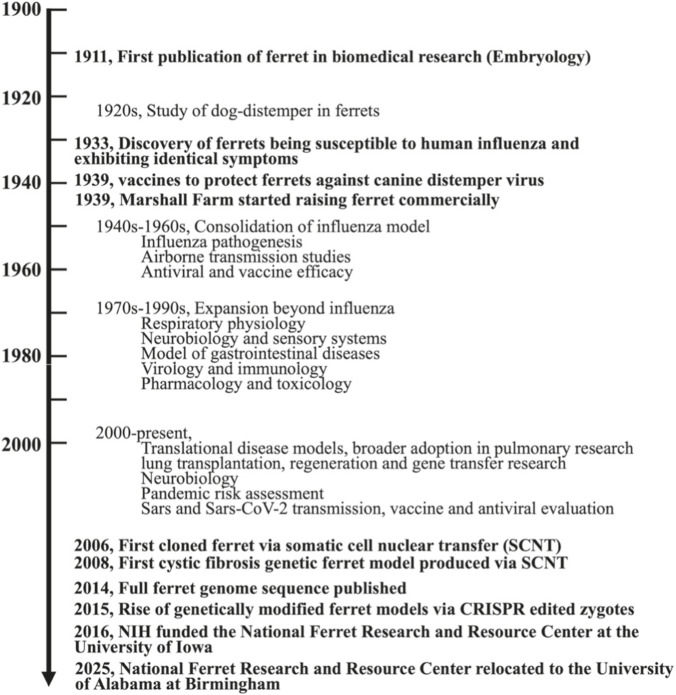
Key milestones in the use of ferrets as biomedical research models.

Ferrets are particularly indispensable for studying influenza virus infection, transmission, and pathogenicity. To support the universal influenza vaccine initiative, the National Institute of Allergy and Infectious Diseases (NIAID)-funded Centers of Excellence for Influenza Research and Response (CEIRR) spearheads efforts to produce monoclonal antibodies, develop assays, and validate cross-reactive and ferret-specific reagents, enabling rigorous and reproducible ferret-based research. Additionally, the CEIRR has established a repository of well-characterized ferret tissues and cell types to further enhance the utility of ferret models (Figure 1) (Albrecht et al., 2018).

Although ferrets have fewer genetic tools and resources for gene function analysis and transgenic model generation than mice, the production of genetically modified (GM) ferrets is feasible (Rosen et al., 2018). The publication of the draft ferret genome sequence and its annotation using the ESEMBL system (Peng et al., 2014) have significantly advanced functional genomic analyses and facilitated the generation of GM ferrets for use in basic biomedical research and human disease modeling. Recognizing the potential of GM ferret models, the National Heart, Lung, and Blood Institute (NHLBI) funded the establishment of the National Ferret Research and Resource Center (NFRRC) at the University of Iowa in 2015. Over the past decade, the NFRRC has generated 31 unique transgenic ferret lines and continues to receive funding from a US Federal Contract (personal communication). Today, the NFRRC collaborates with more than 50 academic institutions and industry partners to advance the use of ferrets and GM ferrets for basic scientific research and as preclinical models for drug discovery and therapeutic evaluation. Figure 1 outlines the key milestones in the use of ferrets as biomedical research models.

## 2 Ferret biology and suitability as experimental animal models

### 2.1 Ferret biology

Ferrets are small-to medium-sized carnivorous mammals with short legs, an elongated body, thick fur, and a long tail averaging 13 cm in length. The most used ferrets in biomedical research laboratories are black sable or “fitch” ferrets, which are characterized by a buff-colored coat with a dark face, feet, and tail markings. Albino ferrets, which have a white coat, red to pink eyes, and pink noses, are also widely used. As obligate carnivores, ferrets require a meat-rich diet and cannot effectively digest vegetable proteins. Adult ferrets typically measure around 50 cm in length and weigh between 0.7 and 2.0 kg, with male ferrets being nearly twice the weight of females. Ferrets are highly social and active animals when awake, but they sleep for up to 18–20 h per day (Pramod et al., 2024). Their average lifespan ranges from 5 to 7 years, though they can live for up to 8–10 years when kept in captivity. They are considered geriatric at 3–4 years of age (Hoppes, 2010).

Ferrets reach sexual maturity within 6–12 months, typically in the spring following their birth. Females can breed by 6–12 months of age, while males are fertile for around 9 months. In ferrets, breeding, gestation, and lactation require a long photoperiod (more daylight hours). Over their lifetime, female ferrets can each produce over 160 offspring. The gestation period lasts for 41–42 days, yielding average litter sizes of eight kits; however, the litter size can range from 1 to 18 (Lindeberg, 2008). Neonatal ferrets (known as “kits”) are altricial, weighing only 8–10 g at birth, and their eyes remain closed until approximately 30 days of age. Kits have voracious appetites and should remain with their mother until at least 6 weeks of age. Weaning is complete by 6–8 weeks of age, with kits reaching approximately 250 g in body weight by this time (Ball, 2006).

### 2.2 Ferrets as experimental models

Animal models are fundamental for scientific research, providing insights into organism biology and facilitating the development of vaccines and therapies. Although mice are the most extensively used experimental animals in biomedical research and the techniques used to create GM mouse models are well established, the significant anatomic and physiological differences between mice and humans limit their relevance in translational medicine, especially for the purpose of modeling complex human diseases and neurological conditions (Gruntman and Flotte, 2022). Although non-human primates (NHPs) are closer analogs to humans, their use is constrained by high costs, ethical concerns, and specialized care requirements (Table 1) (Phillips et al., 2014; Friedman et al., 2017).

**TABLE 1:** Advantages and limitations of ferrets as biomedical research models.

Feature	Ferret	Mouse	Pig	Non-human primate
Body size	Medium	Small	Large	Large
Cost	Moderate	Low	High	Very high
Husbandry requirements	Moderate	Low	High	Very high
Lifespan	8–10 years	2–3 years	10–20 years	Long
Reproductive efficiency	Moderate	High	Moderate	Low
Generation interval	∼6–9 months	∼9–12 weeks	∼1–2 years	Several years
Human-like anatomy	Moderate–High	Low	Moderate–High	Very high
Gyrencephalic brain	Yes	No	Yes	Yes
Human-like neurodevelopment	Moderate–High	Low	Moderate–High	High
Natural susceptibility to human respiratory viruses	Yes	Low	Moderate	Yes
Vomiting reflex	Present	Absent	Present	Present
Availability of genetic tools and reagents	Moderate	Extensive	Moderate	Limited
Ease of genetic engineering	Moderate	Excellent	Moderate	Limited
Translational relevance	High	Moderate	High	Very high
Ethical concerns	Moderate	Low	Moderate	High
Major limitation	Cost, less regents	Limited human relevance	Cost, housing requirements	Cost, ethics, availability

Ferrets are a compelling alternative as they balance practicality with biological relevance. They are more cost-effective, easier to handle, and more practical to maintain than other large non-primate animals, such as dogs and pigs. Although interspecies differences are still a concern, ferrets share numerous key anatomical, physiological, and metabolic similarities with humans, surpassing rodents in this regard. For example, ferrets are naturally susceptible to many human pathogens, and their immune system function more closely resembles that of humans. Extensive studies have demonstrated that the cellular immune response of ferrets to the influenza virus mirrors that of humans (Wong et al., 2019).

The body size of ferrets further enhances their suitability as experimental models. They are relatively small, which is conducive for efficient handling, but they are large enough to enable realistic simulations of human delivery routes, easy sample collection, and sophisticated surgical procedures. Additionally, their short gestation period and large litter size support large-scale experimental designs. Moreover, their much longer lifespan (8–10 years in captivity) compared to rodents allows for longitudinal studies on aging and chronic diseases. Collectively, these features make ferrets invaluable in biomedical research, bridging the gap between rodent models and more costly or ethically challenging NHP models.

Comparisons are generalized and intended to highlight relative strengths and limitations commonly encountered in biomedical research. Specific advantages may vary depending on the disease model and experimental application.

## 3 Ferrets for the study of respiratory infection

In the early 1930s, ferrets were observed to develop influenza-induced rhinitis, leading to the discovery of their natural susceptibility to human influenza viruses (Smith et al., 1933). Since then, research conducted in ferrets has significantly advanced our understanding of influenza virus biology. Unlike mice, ferrets are naturally susceptible to unadapted human influenza virus isolates, including the influenza A subtypes H1N1 (both pre-2009 seasonal and 2009 swine-origin pandemic strains) (Buchman et al., 1995; Munster et al., 2009), H2N2 (Van de Ven et al., 2022), and H3N2 (Bodewes et al., 2013), as well as the influenza B virus (Kim et al., 2009). Ferrets can also be infected with avian influenza A (H5N1) (Zitzow et al., 2002; Boltz et al., 2008), other avian isolates, the H7 and H9 subtypes (Belser et al., 2008; Wan et al., 2008), and swine influenza strains (Shope, 1934).

The clinical course of influenza in ferrets closely resembles that observed in humans, with symptoms such as sneezing, fever, lethargy, and upper (and occasionally lower) respiratory tract infection (Smith and Sweet, 1988; Bodewes et al., 2013). Influenza viruses use the viral attachment protein hemagglutinin to bind to receptors on airway epithelial cells. These receptors are cell surface glycoproteins and glycolipids that terminate with N-acetylneuraminic acid (Neu5A, otherwise known as sialic acid), either in an α2,6-linked (Neu5ACα2,6) or α2,3-linked (Neu5ACα2,3) configuration. Human influenza viruses preferentially bind to Neu5ACα2,6 receptors, whereas avian influenza viruses preferentially bind to Neu5ACα2,3 receptors (Ibricevic et al., 2006).

The distribution of these receptors along the respiratory tract is strikingly similar between ferrets and humans, with a high density of Neu5ACα2,6 in upper airways and both Neu5ACα2,6 and Neu5ACα2,3 in the lower airways (De Lisle et al., 2008). Consequently, ferrets infected with the influenza A strains seasonal H1N1 and H3N2 typically develop mild-to-moderate symptoms with extensive viral replication contained in the upper respiratory tract and limited spread to the lungs, mirroring the course of the disease in humans. Conversely, highly pathogenic influenza viruses with enhanced affinity for the Neu5ACα2,3 receptors can cause severe pneumonia in ferrets and widespread infection through the lungs (Bodewes et al., 2013). For example, one study showed that ferrets inoculated with the reconstructed pandemic 1918 H1N1 virus exhibited higher infectious viral titers in nasal specimens and shed the virus for longer durations than those inoculated with seasonal human H1N1 strains (Pearce et al., 2012). The 1918 H1N1 virus featured efficient viral replication throughout the respiratory tract and in the lungs. One study revealed that two amino acid changes in the hemagglutinin protein were sufficient to switch its receptor specificity from Neu5ACα2,6 (human) to Neu5ACα2,3 (avian), abolishing respiratory droplet transmission between ferrets while retaining the replication efficiency in the upper respiratory tract, as well as its lethality. This finding underscores the importance of preferential binding to the Neu5ACα2,6 receptor for optimal transmission, as well as the critical role of hemagglutinin receptor specificity, in the pathogenesis of influenza (Tumpey et al., 2007).

The similarity of ferrets to humans with respect to influenza susceptibility makes them an invaluable model for evaluating the virulence, pathogenesis, transmissibility, and pandemic risk of influenza viruses (Belser et al., 2018a; Belser et al., 2018b). Recently, ferrets have been used to assess the disease characteristics and transmission of a novel avian influenza A virus from a human case in Texas (A/Texas/37/2024 virus). The study found that this strain caused severe disease, with 100% lethality in all infected ferrets (N = 6), and that it spread efficiently by direct contact with other ferrets (N = 3). However, its transmission via respiratory droplets was less efficient, with no spread observed in healthy ferrets housed in adjacent enclosures sharing air but without direct contact. The ability of the virus to spread in a fomite-mediated transmission model (via contaminated surfaces) was also tested. The results showed that one in three healthy ferrets exposed to the contaminated surfaces became infected. For comparison, the seasonal influenza virus strain infected all three previously healthy ferrets in this test model. These results suggest that while the A/Texas/37/2024 strain poses a significant risk, unlike the seasonal influenza virus, it is not yet capable of efficient transmission among humans via respiratory droplets (Pulit-Penaloza et al., 2024a). The risk of another highly pathogenic avian influenza A (H5N1) virus isolated from a human case in Chile has also been evaluated in ferrets. The virus caused fatal disease in ferrets and was transmitted via direct contact between co-housed ferrets. However, it did not exhibit efficient transmission via respiratory droplets or fomite-mediated transmission (Pulit-Penaloza et al., 2024b).

Beyond influenza, ferrets have proven invaluable for modeling the human disease course of several other human respiratory viruses, including respiratory syncytial virus (Stittelaar et al., 2016), parainfluenza virus, and severe acute respiratory syndrome (SARS)-coronavirus 1 (SARS-CoV1) and SARS-CoV2 (Ciurkiewicz M et al., 2022). Studies of SARS-CoV2 infection in ferrets have revealed high transmission rates through direct contact and airborne routes, similar to humans (Kim et al., 2020; Richard et al., 2020), but infected ferrets generally develop mild symptoms, with viral replication restricted to the upper airways (Ciurkiewicz et al., 2022). Another study showed that aged ferrets can develop high viral loads, prolonged nasal virus shedding, severe pneumonia, and clinical signs, resembling those observed in terminally ill patients with coronavirus disease 2019 (COVID-19) (Kim et al., 2022).

Ferrets have also been used to model the transmission of tuberculosis. *Mycobacterium tuberculosis* (*M. tuberculosis*) is a persistent pathogen that has long plagued humanity. Tuberculosis remains a leading cause of death resulting from a single pathogen in humans. Ferrets are natural hosts of *Mycobacterium bovis*, which acts as the transmission vehicle for bovine tuberculosis (de Lisle et al., 2008; McCallan et al., 2011). Notably, it has been shown that intratracheal infection with a high dose of *M. tuberculosis* in ferrets resulted in clinical signs and pathological features akin to tuberculosis in larger animal models. Acutely infected ferrets efficiently transmitted *M. tuberculosis* to co-housed *M. tuberculosis*-naïve sentinel animals. Most of the co-housed ferrets were positive for *M. tuberculosis* in nasal washes, and some of them developed variable disease symptoms similar to those reported in humans exposed to a patient with active tuberculosis in a closed setting (Gupta et al., 2022).

The susceptibility of ferrets to human pathogens is largely attributed to the presence of shared receptors, but it is also attributed to their great anatomical similarities to humans. For instance, like humans, ferrets have a long trachea, dense submucosal glands in the tracheobronchial walls, and a human-like number of terminal bronchiole generations. These features enable detailed studies of the dynamics of infection and the resulting effects in different regions of the respiratory tract. The body size of ferrets enables straightforward monitoring of clinical parameters, such as temperature, pulse, and respiratory rate, offering valuable insights into disease progression. All of these characteristics, alongside the immunological similarities between ferrets and humans, make ferrets an indispensable model and a reliable platform for studying respiratory infections and advancing therapeutic and vaccine development. Furthermore, ferrets can become immunocompromised by the oral administration of a combination of immunosuppressive compounds used in transplantation patients, including tacrolimus, mycophenolate mofetil, and prednisolone (Stittelaar et al., 2016) or methylprednisolone, azathioprine, and cyclosporine (Tang et al., 2023). Given the inherent difficulties in studying antiviral efficacy in immunocompromised patients, immunocompromised ferrets offer an attractive alternative for studying the efficacy of such therapies in preclinical studies (Van der Vries et al., 2013; Stittelaar et al., 2016).

## 4 Modeling human pulmonary conditions

The structural and functional similarities between the respiratory systems of ferrets and humans highlight the valuable utility of ferrets for modeling various human pulmonary conditions. Ferrets have also been used in preclinical studies to evaluate pulmonary drug delivery, and they have shown potential as models for assessing pulmonary toxicity caused by environmental agents. In this section, we describe what is known about the use of ferrets as models for studying human pulmonary conditions.

### 4.1 Chronic obstructive pulmonary disease (COPD)

COPD is a progressive lung disease with limited treatment options. Ferrets exposed to cigarette smoke develop clinical features of COPD, including chronic bronchitis and bronchiolitis, whereas most mouse models of COPD primarily manifest with emphysematous disease. The changes in gene expression in ferrets with cigarette smoke-induced COPD are consistent with those observed in humans with COPD, suggesting that ferret models of COPD are well-suited for studying the pathophysiology and therapeutic interventions for this disease (Raju et al., 2016; Hussain et al., 2022).

### 4.2 Idiopathic pulmonary fibrosis (IPF)

IPF is a complex and heterogeneous genetic disease that involves mucociliary dysfunction of the peripheral airways (Schwartz, 2016). Mutations in *MUC5B* (encoding mucin 5B) are implicated in the pathogenesis of IPF. *MUC5B* mRNA and protein are detected in the bronchoalveolar epithelia, with the characteristic honeycomb cysts of IPF (Evans et al., 2016). Unlike rodent models of IPF, in which bleomycin-induced fibrosis failed to sustain long-term airway remodeling, bleomycin-treated ferrets exhibited persistent fibrosis lasting for up to 22 weeks. They developed key pathologic features, including proximalization of the distal airways, abnormal distribution of MUC5B-expressing cell types, and MUC5B-positive cystic structures in the distal lung, resembling the honeycombing observed in humans with IPF (Peabody Lever et al., 2024).

### 4.3 Acute lung injury and acute respiratory distress syndrome (ARDS)

Acute lung injury is a type of acute respiratory failure that occurs when the lungs are suddenly damaged, impairing gas exchange (Johnson and Matthay, 2010). ARDS is a severe condition that is characterized by widespread lung inflammation and damage to the alveolar–capillary barrier, culminating in severe breathing difficulties (Rubenfeld et al., 2005). Acute lung injury and ARDS are triggered by various factors, such as infections (viral and bacterial), trauma, and toxin exposure. Ferrets can develop such conditions, and their manifestations are similar to those observed in humans, making ferrets a valuable model for preclinical respiratory inflammation studies, particularly through exposure to lipopolysaccharide, mechanical ventilation, or other injurious stimuli that replicate the human inflammatory and repair responses (Khoury et al., 2022).

SARS-CoV2 can lead to ARDS in severe cases of COVID-19. In such cases, ARDS is characterized by pathological changes, such as diffuse alveolar damage in the lungs, fluid accumulation in the lungs, and below-normal levels of oxygen in the blood (Wu et al., 2020). Although ferrets with SARS-CoV2 infection usually show mild disease with viral replication restricted to the upper airways, aged ferrets (≥3 years old) show severe lung inflammatory cell infiltration and more clinical symptoms than juvenile (≤6 months) and young adult (1–2 years) ferrets (Kim et al., 2022).

### 4.4 Pulmonary hypertension

Pulmonary hypertension is characterized by elevated blood pressure in the pulmonary arteries. It most commonly occurs secondary to underlying respiratory diseases that cause airway inflammation and obstruction. In humans, chronic bronchitis and infectious agents, such as *Mycoplasma pneumoniae*, are recognized contributors to pulmonary hypertension. Similarly, *Mycoplasma* spp. have been reported as potential causative microbial agents in ferrets with respiratory disease. Cases of chronic bronchitis and *Mycoplasma*-induced pneumonia leading to the development of pulmonary hypertension in domestic ferrets have been reported, underscoring their relevance as a model for studying the interplay between airway diseases and vascular complications in the lungs (Day et al., 2024).

### 4.5 Lung cancer

A previous study showed that ferrets exposed to a tobacco carcinogen [1-(3-pyridyl)-1-butanone (4-(N-methyl-N-nitrosamino)-1-(3-pyridyl)-1-butanone] developed a range of preneoplastic lesions, including squamous metaplasia, dysplasia, atypical adenomatous hyperplasia, and invasive tumors, including squamous cell carcinoma, adenocarcinoma, and adenosquamous carcinoma, similar to those commonly seen in humans. Therefore, ferrets may be a simple and highly relevant non-rodent model for studying biomarkers and molecular targets for the prevention, detection, and treatment of lung carcinogenesis in humans (Aizawa et al., 2013).

### 4.6 Pulmonary drug delivery

Ferrets are a useful model for studying pulmonary drug delivery via powder aerosols. Such research allows the efficacy of formulations and devices, as well as the deposition of drugs in clinically relevant regions of the lungs, to be evaluated (Kuehl et al., 2019). Moreover, ferrets have been used as animal models to evaluate gene therapy for pulmonary diseases. This approach faces significant challenges owing to the lung’s natural defense mechanisms against foreign invaders, including gene transfer agents. These defenses include a thick layer of mucus, mucociliary clearance (MCC) that actively removes foreign substances from the respiratory tract through ciliary function and airway surface fluid secretion, and the presence of tight junctions between airway epithelial cells. Together, these barriers hinder the efficient delivery of therapeutic vectors to target cells within lung tissues.

Ferrets, with their anatomical and physiological similarities to humans with respect to the respiratory system and their ability to mount more robust immune responses than mice, serve as excellent preclinical models for evaluating airway gene transfer strategies. Among the airway transduction vectors under investigation, recombinant adeno-associated virus (AAV) 2.5T vector (rAAV2.5T) has shown promise and is currently undergoing a phase I clinical trial for cystic fibrosis (CF) lung disease (NCT06526923). Developed through the directed evolution of the AAV capsid library in human airway epithelial cultures, rAAV2.5T has demonstrated efficient gene transfer for restoring transepithelial ion transport in polarized airway epithelial cultures derived from CF patient donors (Excoffon et al., 2009). Preclinical studies in both wild-type and CF ferrets have validated the *in vivo* efficacy of rAAV2.5T, demonstrating that this vector can enable functional therapeutic gene expression in the lungs (Excoffon et al., 2024a; Excoffon et al., 2024b). Ferrets have also been used to test the dosing and repeat dosing of rAAV2.5T in the lungs via intratracheal instillation. Successful transduction (reporter expression) was observed from the second dose in juvenile ferrets at 2 months of age who previously were administered the same vector at 1 month of age (Tang et al., 2020b), as well as in adult ferrets treated with two doses 5 months apart (Tang et al., 2024). The feasibility of repeat dosing to the lungs of immunocompetent and immunocompromised ferrets has also been reported (Tang et al., 2023).

## 5 Modeling gastrointestinal diseases

*Helicobacter pylori* (*H. pylori*) is a bacterium associated with infections of the stomach and small intestine in humans. Approximately 15% of people infected with *H. pylori* will develop duodenal and/or gastric ulcers (Leontiadis et al., 2009). Chronic *H. pylori* infection is also associated with the development of gastric cancers, including adenocarcinoma and gastric mucosa-associated lymphoid tissue (MALT) lymphoma (Parsonnet et al., 1991; Parsonnet et al., 1994; Fischbach et al., 2004; Yao et al., 2023).

In 1985, a helicobacter-like organism was isolated from the margins of a duodenal ulcer in a ferret and was later named *Helicobacter mustelae* (Fox et al., 1986; Fox et al., 1989). Gastric infection with *H. mustelae* is extremely common in domestic ferrets, with gastritis and peptic ulcers routinely reported in ferrets colonized with this bacterium (Fox et al., 1991a; Fox et al., 1991b). In one report, all ferrets with chronic gastritis tested positive for *H. mustelae*, while SPF ferrets, which were free of *H. mustelae*, showed no signs of gastritis, gastric ulcers, or detectable immunoglobulin G antibody (Fox et al., 1990).

Experimental inoculation and other studies have also established *Helicobacter*-induced gastritis in ferrets, which are considered as robust models for understanding the epidemiology, pathogenesis, and treatment of *Helicobacter*-induced gastritis (Patterson et al., 2003). Notably, *H. mustelae* remains the only *Helicobacter* species other than *H. pylori* that is known to cause gastric ulceration and cancer in its natural host (Whary and Fox, 2004; O'Toole et al., 2010). Adenocarcinoma and MALT lymphoma have also been observed in ferrets infected with *H. mustelae* (Erdman et al., 1997; Fox et al., 1997), making ferrets an attractive model for studying the pathogenesis and treatment of gastric cancers in humans.

In addition to gastrointestinal disease research, ferrets are considered a valuable animal model for research on nausea and vomiting (Goineau et al., 2013). Researchers have used ferrets to investigate emetic mechanisms and evaluate anti-emetic drugs with high translational relevance to clinical applications, particularly in understanding chemotherapy-induced nausea and vomiting. Specifically, ferrets have been used to evaluate the emetic potential of drugs (Soref et al., 2012) and to develop anti-emetic medications, such as those prescribed to patients with cancer undergoing chemotherapy (Duffy et al., 2012).

## 6 Neurobiology and behavioral studies

Ferrets have also emerged as valuable models for research on sensory neurobiology owing to their well-characterized sensory systems. They have been used to investigate higher-level visual processes previously identified in primates (Lempel and Nielsen, 2019), as well as visual cortex plasticity and cross-modal sensory integration (Medina et al., 2023). These studies have provided critical insights into how sensory experiences shape neural development. Additionally, the human-like hearing range of ferrets and their ability to be trained make ferrets particularly useful for auditory and multisensory neuroscience studies (Joshi et al., 2024; Norris and Bizley, 2024).

The neocortex, which is the largest part of the cerebral cortex, plays a central role in human cognition and behavior. Despite their relatively small body size, ferrets possess a well-developed neocortex with a gyrencephalic (folded) brain structure, which closely resembles the human brain. In comparison, rodents have a lissencephalic (smooth) brain. This anatomical similarity between ferrets and humans makes ferrets a relevant model for studying cortical development and organization. Neurogenesis in ferrets lasts for ∼5 weeks (Jackson et al., 1989; Reillo and Borrell, 2012), compared to 110 days in humans and only 9 days in mice (Stepien et al., 2021). Ferrets are born with an immature brain, and many key neurodevelopmental processes, including cortical folding (gyrification), neuronal migration, and synaptic maturation, continue well into early postnatal life (Sawada and Watanabe, 2012). These extended developmental timelines make ferrets invaluable models for investigating the development and evolutionary expansion of the neocortex, as well as postnatal neurogenesis and neural plasticity (Gilardi and Kalebic, 2021).

In addition to possessing a gyrencephalic cortex, ferret brains exhibit several structural features that more closely resemble those of humans than rodents. These include diverse astrocyte subtypes and subcortical U-fibers, both of which are largely absent or poorly developed in mice (Tai et al., 2024; Yoshino et al., 2020). Recent studies have further demonstrated that astrocytes play an important role in cortical folding, as reducing astrocyte numbers suppresses gyrus formation in the developing ferret cortex, whereas increasing astrocyte numbers can induce gyrus-like protrusions in the normally lissencephalic mouse brain (Shinmyo et al., 2022). These characteristics make ferrets a valuable model for investigating the cellular and molecular mechanisms underlying higher-order brain organization, cortical development, and neurological disorders that cannot be adequately studied in traditional rodent models.

Ferrets have also been used to model human neurological conditions, injuries, and neurodevelopmental malformations, particularly those involving gyrencephalic brain features or sensory systems. For example, ferrets have been used to study epilepsy (Majkowski, 1983) and traumatic brain injury (TBI) (Schwerin et al., 2018). Cortical dysplasia, which is a neurodevelopmental defect caused by impaired neuronal migration and is associated with epilepsy and intellectual disability, has been modeled in ferrets through injection of the anti-mitotic drug methylazoxymethanol (Noctor et al., 1999; Hasling et al., 2003). Although studies on TBI in rodents have provided valuable insights into the effects of injury and subsequent recovery, ferrets offer greater clinical relevance owing to their neuroanatomical similarities to humans, making them particularly useful for researching the effects of TBI on behavior, memory, and cortical connectivity (Schwerin et al., 2017). Furthermore, their amenability to behavioral training and their compatibility with preclinical magnetic resonance imaging (MRI) facilitate longitudinal studies of brain injuries, therapeutic interventions, and recovery processes (Neal et al., 2007; Barnette et al., 2009).

Ferrets are also highly sociable animals, with complex, trainable behaviors (Obasa et al., 2022), making them valuable for research on learning, memory, and motor control. For instance, ferrets have been used to assess neurodevelopmental disorders and to evaluate the effects of pharmacological agents on cognition and behavior (Goodfellow et al., 2024). Their sociability and tendency for environmental exploration further position them as relevant models for studying social and affective behavior (Jimenez et al., 2023).

## 7 Reproduction biology studies

Domestic ferrets, a seasonally polyoestrous species, also serve as important models for studying reproductive biology. Early research on ferret reproduction primarily focused on their seasonal breeding cycle, mechanism of induced ovulation, and the impact of the photoperiod (day length) on reproductive activity. These studies have been instrumental in uncovering the environmental and physiological factors that influence seasonal reproductive cycles, puberty, and ovulation (Bakker et al., 2001; Pramod et al., 2024). Advances in assisted-reproductive technologies in ferrets, particularly research on oocyte maturation, fertilization, embryonic development, and embryo transplantation, have further expanded their applications as models for reproductive biology research (Li et al., 2002). A landmark achievement in this area was the successful cloning of a ferret via somatic cell nuclear transfer (SCNT) in 2006 (Li et al., 2006). This breakthrough not only underscored the value of ferrets as models for reproductive biology, but it also paved the way for creating GM ferret models, further enhancing their utility in biomedical research (Table 2).

**TABLE 2:** Genetically modified ferret models and their applications in biomedical research.

Model	Genetic modification	Research area	Major application
*CFTR* ^KO^	*CFTR* disrupted	Cystic fibrosis	Disease pathogenesis
*CFTR* ^G551D^	Disease-causing mutation (gating mutation)	Cystic fibrosis	Disease pathogenesis, modulator testing, gene therapy
*CFTR* ^F508del^	Disease-causing mutation (protein processing mutation)	Cystic fibrosis	Disease pathogenesis, modulator testing, gene therapy
*CFTR* ^Ex16(lsl)^	Lox P sites knock-in flanking Exon 16	Cystic fibrosis	Conditional knockout
*CFTR* ^int-eGFP(lsl)^	Exon-trap eGFP reporter in intron 1	Cystic fibrosis	Conditional functional restoration
*ROSA* ^mTmG^	mTmG reporter knock-in in *ROSA* locus, a genome safe harbor	Airway biology, gene transfer reporter	Gene transfer assessment, cell fate mapping
*FOXI1* ^KO^	*FOXI1* disrupted	Airway biology	Ionocyte function
*FOXI1* ^CreERT2^	CreERT2 knock-in at the untranslated region of the last exon	Airway biology	Ionocyte lineage studies
*Muc5B* ^CreERT2^	CreERT2 knock-in at the untranslated region of the last exon	Airway biology	Goblet cell studies
*SFTPC* ^CreERT2^	CreERT2 knock-in at the untranslated region of the last exon	Lung biology	Alveolar cell studies
*ASPM* ^KO^	*ASPM* disrupted	Neurobiology	Human microcephaly studies
*DCX* ^KO^	*DCX* disrupted	Neurobiology	Human brain development disorders

## 8 Development of GM ferret models

### 8.1 Ferrets as a model of CF

The advent of genetic engineering techniques for domestic ferrets has dramatically expanded their utility as model organisms for studying the molecular basis of human diseases that cannot be adequately replicated in GM mouse models. One notable example is CF, a genetically inherited recessive disease caused by defects in *CFTR*, which encodes the cystic fibrosis transmembrane conductance regulator (CFTR) protein (Rommens et al., 1989). CF is a progressive disease that affects multiple organs. However, the primary cause of mortality is pulmonary failure due to chronic bacterial infection in the lungs (Stoltz et al., 2015).

Although mouse and rat models of CF reproduce some aspects of CF in humans, they fail to reproduce the hallmark phenotype of spontaneous bacterial colonization in the lungs. The first GM ferret was developed for modeling human CF. It was created by SCNT with *CFTR*-disrupted (knockout; KO) ferret fibroblasts as nuclear donors. *CFTR* disruption was achieved through AAV vector-mediated gene targeting (Sun et al., 2008). These *CFTR*-KO ferrets closely recapitulated the phenotypes of human CF in multiple organs, including the lungs (Sun et al., 2014). However, CF ferrets exhibit a high incidence of severe meconium ileus (MI) at birth, affecting approximately 75% of kits, compared with ∼15% of infants with CF (Gaillard et al., 1996; Oppenheimer and Esterly, 1962). Consequently, survival of CF kits is markedly reduced. Furthermore, CF kits born without severe intestinal disease rapidly develop polymicrobial lung infections within the first weeks of life without antibiotic intervention (Sun et al., 2010; Sun et al., 2014). These complications initially posed significant challenges for the breeding and maintenance of *CFTR*-knockout ferrets, although subsequent advances in husbandry and veterinary care have substantially improved their survival and utility as disease models.

Later, advances in clustered regularly interspaced short palindromic repeats (CRISPR)-based gene-editing techniques significantly improved the production efficiency of GM ferret models, allowing for precise genome manipulation in zygotes (single-cell embryos). Using CRISPR, advanced CF ferret models expressing dysfunctional CFTR^F508del^ and CFTR^G551D^ have been developed (Sun et al., 2019; Yan et al., 2022; Evans et al., 2024). These ferret models of CF correspond to two common *CFTR* defects in humans, offering higher translational relevance than the CFTR-KO model. *CFTR* ^F508del^ is the most common CF genotype, accounting for approximately 70% of all CF alleles, while *CFTR* ^G551D^ is present in 4%–5% of patients with at least one affected allele. F508del CFTR is a misfolded mutant that fails to reach the surface of epithelial cells, and G551D is a gating mutation that impairs the ion channel function of the CFTR protein.

In CF ferret models with *CFTR* ^F508del^ or *CFTR* ^G551D^, disease progression closely mimics that observed in CF patients with these genotypes. Therefore, these models can be used to evaluate the effects of pharmaceutical interventions, such as CFTR modulators. CFTR modulators can restore the function of mutant CFTR proteins; however, this treatment option is CFTR genotype-dependent (Taylor-Cousar et al., 2023). For instance, the CFTR potentiator ivacaftor (VX-770) has proven effective in ferrets with *CFTR* ^G551D^. CF starts *in utero* and impacts postnatal health. One study showed that administering VX-770 during and after gestation led to higher survival rates and normal growth after birth, alleviating the challenges associated with rearing severely ill animals (Sun et al., 2019). Importantly, the onset of CF in this model is pharmaceutically manageable, and discontinuing treatment at any age leads to reinitiation of CF in the pancreas, gut, and lungs. Ferret models of CF have also been used for basic research on the pathogenesis of CF and to evaluate CFTR biology in different organs, as well as in preclinical therapeutic studies of gene therapy (Excoffon et al., 2024b). Ferrets with *CFTR* ^F508del^ and *CFTR* ^G551D^ have also been instrumental in exploring innovative therapeutic genome-editing approaches, which aim to permanently correct *CFTR* mutations at the genome level (Yan et al., 2022). Such approaches could eliminate the need for repeat dosing required by conventional gene-addition approaches (Tang et al., 2020a).

### 8.2 Ferrets as a model for human neurological disorders

GM ferrets have been developed to model human neurological disorders that cannot be effectively modeled in mice. One such example is the use of ferrets to model microcephaly. KO of *ASPM* (which encodes the abnormal spindle-like microcephaly-associated protein) in ferrets was the second gene KO model developed in ferrets to study human disease, following the creation of CF ferret models. In contrast to *ASPM* KO mice, which showed a reduction in brain size of only 10%, *ASPM* KO ferrets exhibited severe microcephaly characterized by a decrease of up to 40% in brain weight. The absence of ASPM expression in ferrets led to premature detachment and migration of outer radial glial cells from the ventricles, resulting in deficits in the cerebral cortex (Johnson et al., 2018). *ASPM* KO ferrets demonstrated a reduced cortical surface area without a significant change in cortical thickness, closely mirroring the phenotype observed in human patients with microcephaly.

Doublecortin (DCX) is essential for neural progenitor cell proliferation and radial glial fiber extension. In humans, the *DCX* gene is located on the X chromosome, and as such, its mutations exhibit sex-specific phenotypes. Hemizygous *DCX* mutations in males cause classical lissencephaly, while heterozygous mutations in females result in subcortical band heterotopia (SBH). *DCX* KO ferrets effectively recapitulated the key features of lissencephaly or SBH in humans and displayed sex-specific manifestations (Wang et al., 2024).

In addition to germline genetic engineering, complementary approaches such as *in utero* electroporation (IUE) (Kawasaki et al., 2012), IUE combined with CRISPR/Cas9-mediated genome editing (Tsunekawa et al., 2016; Shinmyo et al., 2017), and piggyBac-based transgenesis (Shinmyo et al., 2022; Hamabe-Horiike et al., 2021) have substantially expanded the utility of ferrets in neuroscience research. These techniques enable rapid and efficient manipulation of gene expression in the developing ferret brain without requiring the generation of new transgenic lines, thereby accelerating mechanistic studies of cortical development and neurological disease. Applications of these approaches have provided important insights into the molecular regulation of cortical folding, lineage specification, astrocyte diversity, and higher-order brain organization in this gyrencephalic mammalian model (Yoshino et al., 2026).

### 8.3 Application of the cre/Lox recombination system in ferrets

The Cre/Lox recombination system is a powerful and versatile experimental tool that is widely used in mammalian genetics and cell biology research (Kuhn and Torres, 2002). In mice, a common approach pairs two transgenic mouse lines to enable precise spatial and temporal control of gene expression, lineage tracing, and conditional gene KO or activation. With this approach, one transgenic line typically carries a LoxP-STOP-Lox (LSL) sequence-associated transgene or reporter integrated at the ROSA26 locus, a genome “safe harbor” site, or conditional gene KO at an endogenous locus. An example is the *ROSA* ^mTmG^ line (Jackson Laboratory, IMSR_JAX:007676) with the Cre recombinase (Cre)-responsive fluorescence-conversable reporter (Muzumdar et al., 2007). The second line, referred to as Cre driver, expresses the CRE-ERT2 fusion protein, which functions to mediate recombination between LoxP sites and requires tamoxifen-induced nuclear transport (Hayashi and McMahon, 2002). The expression of CRE-ERT2 in Cre drivers can be ubiquitous or cell/tissue-specific.

A similar recombination system has been adapted for use in ferrets. Like the *ROSA* ^mTmG^ mouse line, *ROSA* ^mTmG^ ferrets carry a LoxP-tdTomato-STOP-LoxP-eGFP (LSL-reporters) driven by the ubiquitous CAG promoter at the genomic safe harbor. Upon excision of the LSL tdTomato reporter, eGFP expression is activated, allowing for the precise visualization of cellular processes that are responsive to Cre-mediated recombination (Yu et al., 2019). Several cell-specific Cre driver lines have been developed in ferrets, enabling lineage tracing and targeted gene manipulation. The *ROSA* ^mTmG^ ferret, in combination with Cre drivers, constitutes the only model system available for lineage tracing studies in a large animal.

One notable application is *FOXI1-Cre* ^*ERT2*^ ferrets, in which Cre-mediated recombination is restricted to FOXI1-expressing cells. Crossing ROSA^mTmG^ ferrets with *FOXI1-Cre* ^*ERT2*^ ferrets allows for genetic labeling of pulmonary ionocytes in the respiratory system, which are visualized by red-to-green fluorescence conversion. Breeding these ferrets with *CFTR* conditional KO (*CFTR* ^L/L^) ferrets, which carry a Cre-excisable *CFTR* exon 16, yields the *ROSA* ^mTmG^/*FOXI1-Cre* ^*ERT2*^/*CFTR* ^L/L^ compound model. This model facilitates research into the functions of ionocytes in anion secretion, absorption, and MCC under the condition of precise and cell-specific *CFTR* deletion, providing critical insights into the roles of pulmonary ionocytes in the pathogenesis of CF (Yuan et al., 2023).

## 9 Challenges

The selection of an appropriate animal model for biomedical research requires careful consideration, as each species presents unique advantages and limitations that vary with the research context. Interspecies differences often constrain the direct translatability of animal studies to humans. However, ferrets have demonstrated their utility in various biomedical research fields, striking an impressive balance between biological relevance and practicality; however, several challenges still limit their broader application, which are described in the following sections.

### 9.1 Cost and husbandry

Ferrets are significantly more expensive to maintain than rodents, with the higher costs resulting from their housing, specialized diets, and veterinary care. Moreover, their husbandry requires dedicated facilities and trained personnel, posing further logistical and financial challenges. Additionally, research involving ferrets is subject to stricter regulatory and ethical guidelines compared to the use of rodent models and smaller model organisms, which complicates their use in scientific experiments.

### 9.2 Environmental and health considerations

Experimental ferrets thrive only under specific environmental conditions, requiring controlled temperatures, humidity, and ventilation in research facilities. Their susceptibility to human pathogens, such as the influenza virus and other respiratory infection-causing pathogens, necessitates rigorous health monitoring and management protocols. This poses additional challenges, as a disease outbreak can compromise the integrity of ongoing experiments.

### 9.3 Genomic data limitations

Although the draft ferret genome is available and CRISPR-based genome editing in ferret zygotes has been established, the lack of well-annotated genomic sequence information remains a significant limitation to the widespread utility of ferrets in experimental research. Inadequate genomic resources hinder detailed genetic analyses and the efficient development of GM ferret models, particularly compared to the more extensively characterized genomes of rodent species.

### 9.4 Immunological reagents and knowledge gaps

Ferret-specific reagents, particularly T- and B cell-specific antibodies for phenotyping, are limited. Additionally, the characterization of ferret immunoglobulin subclasses and Fc receptors is incomplete. These gaps constrain immunological research to comprehensively understand the innate and adaptive immune responses and the development of ferret models for immune-related disorders.

## 10 Moving forward and future directions

Despite the barriers, significant progress has been made in overcoming many limitations associated with the use of ferret models in experimental research. Advances in sequencing technologies, genome annotation, and genome-wide gene expression analyses, including single-cell RNA sequencing and spatial transcriptomics (Bilgic et al., 2023; Yuan et al., 2023), have enhanced our understanding of ferret genetics and enabled precise genetic engineering. Comparative transcriptomics analyses between human diseases, ferret models, and host transcriptome studies following viral infection in various ferret tissues are providing deeper insights into phenotypic characterization, immune responses, and pathway activation (Cross et al., 2018; Lee et al., 2021).

Advancements in developing ferret-specific antibodies, cytokine assays, and other immunological reagents have expanded our research capabilities (Albrecht et al., 2018; Wong et al., 2019). Additionally, validation of the compatibility of existing cell marker antibodies to cross-react with ferret lymphocytes and other white blood cells that contribute to the body’s defense, has further facilitated immunological studies (Hundakova et al., 2022). The development of new GM ferret models tailored to specific diseases, such as the alpha-1 antitrypsin deficiency ferret model for lung and liver diseases (He et al., 2022) and the newest ferret model of CF that enables visualization of CFTR-expressing cells and targeted functional rescue (Yuan et al., 2025), has significantly expanded the toolbox for studying disease pathogenesis and testing therapeutics. Improved breeding and husbandry protocols for managing sick animals have also further enhanced their practical use in basic research and preclinical studies (Sun et al., 2019).

Interinstitutional efforts and resource-sharing initiatives, such as the collaboration led by the National Institutes of Health (NIH)-funded CEIRR and NFRRC, are essential for accelerating the development and accessibility of ferret-related tools, resources, and data. These initiatives will encourage more researchers to incorporate ferret models into their existing research and promote the development of new models within the diverse framework of translational research. The CEIRR has expanded the availability of monoclonal antibodies, cross-reactive and ferret-specific reagents, and validated assays (Albrecht et al., 2018). In parallel, the NFRRC has cataloged over 169 antibodies tested in ferrets in collaboration with CEIRR (personal communication). These advances are enhancing the rigor, reproducibility, and overall utility of ferret-based research, as reflected in its growing impact. With continued advances in genomic technologies, immunological tools, and model development, ferrets are well-positioned to become even more valuable as translational research models. By addressing existing challenges and fostering collaborative efforts, ferrets are likely to play an increasingly important role in advancing our understanding of human diseases and in developing innovative therapeutics, surpassing other large-animal models, such as dogs, rabbits, and pigs.
